# Supplementary material for: Are carbon emissions trading and green financial instruments synergistic? -Comprehensive quantitative research based on content analysis
Source: PLoS One. 2024 Mar 7;19(3):e0298601. doi: 10.1371/journal.pone.0298601 (PMC10919601; doi:10.1371/journal.pone.0298601)
Supplement: S1 Data — (ZIP) [file pone.0298601.s001.zip › Data/地级市Z_F工作报告.zip!/║■▒▒/╗╞╩»╩╨/└┤╫╘╣1⁄2╓┌║┼í╛┬φ┐╦╩2╛▌═°í┐_2019.docx]

各位代表:

现在，我代表市人民政府向大会报告工作，请予审议，并请市政协各位委员和列席人员提出意见。

一、2018年工作回顾

一年来，面对国内外形势错综复杂、经济下行压力加大的严峻挑战，在省委省政府和市委的正确领导下，我们坚持以习近平新时代中国特色社会主义思想为指导，认真贯彻落实党的十九大和习近平总书记视察湖北重要讲话精神，以打好三大攻坚战、推进高质量发展为主题主线，坚定不移调结构、换动能、转方式、促协调、固底板，全市经济社会发展呈现稳中有进、逆势而上、提质增效的良好态势，朝着全面建成小康社会目标又迈进了坚实一步。

——稳的基础在巩固。经济运行总体稳定在合理区间，除地方公共财政预算收入增幅由于落实国家减税降费政策等原因没有完成外，其他主要经济指标均较好完成市十四届人大三次会议确定的目标。地区生产总值增长7.8%；规模以上工业增加值增长9%；固定资产投资增长11.1%；全市社会消费品零售总额增长11.1%；地方公共财政预算收入增长5.3%；外贸出口增长9%；新增城镇就业人口5.4万人，城镇登记失业率控制在3.2%以内。

——进的势头在延续。工业技改投资增长17.7%，8家企业入围全省百强、居全省第二。新增规模以上工业企业61家，9个产业入选省重点成长性产业集群，东贝压缩机产销超过3000万台、单个品牌全球第一。黄石在国家产业转型升级示范区考核中名列第二。项目建设“承诺预办、先建后验”在国务院第五次大督查中被通报表扬。在全省市州率先开通跨境电商业务，外贸进出口总额37.1亿美元、增长10%，总量全省第二，黄石连续9年被评为全国外贸百强城市，国务院批复同意黄石港口岸扩大开放棋盘洲港区。劲牌公司吴少勋入选“改革开放40周年全国百名杰出民营企业家”。阳新县脱贫摘帽目标有望如期实现，黄石、大冶同时入选新时代中国全面建成小康社会优秀城市。大冶市全国百强进至77位,获批全国首批创新型县（市）。

——高质量发展的格局在形成。发展的含金量、含新量、含绿量大幅提升。地方税收占财政收入比重接近70%、提高近5个百分点。57家企业入选全省隐形冠军示范、培育企业，新增高新技术企业60家，高新技术产业增加值增长12.4%，占GDP比重提高2.4个百分点。电子信息、生物医药、高端装备制造、新能源汽车四大产业对工业增长贡献率超过30%。黄石大冶湖高新区晋升为国家高新区，我市成为全省第三个同时拥有国家级经开区和高新区的城市。新登记市场主体2.8万户。单位产值能耗下降3%。

——复兴黄石的有利条件在汇聚。大冶湖新区建设蹄疾步稳，一座临港临空、产城融合的长江新城加速崛起。随着长江中游第一条公铁水多式联运线路正式开通，投资200亿元的新港重科引进落户，新港已成为黄石最具发展活力的引擎。团城山综合客运枢纽站建成使用，直达上海的“劲牌号”高铁开通运行，黄石（铁山）至武汉（光谷）一级公路路基全线贯通，亚洲第一、紧邻黄石的湖北国际物流核心枢纽开工建设，黄石交通枢纽地位更加凸显。中国工业互联网创新发展大会在我市召开，黄石被确定为永久会址城市。城市的聚集力、辐射力、吸引力显著提升，新落户2.12万人，黄石正在成为全国性的打工目的地城市。

——人民群众的获得感、幸福感、安全感在增强。办成了一系列大事、喜事、要事、盛事。成功承办全省县域经济现场会、第十五届省运会、中国（黄石）第二届地矿科普大会、中国铜加工产业年度大会，全面展示了黄石城市转型的新形象、新成就。常态化开通工业旅游专线火车，铜绿山古铜矿遗址入围第二批国家工业遗产名单，城市魅力更加彰显。成功创建国家森林城市，国家园林城市通过复查，城市环境面貌明显改善，蓝天、碧水、绿地越来越多。民生支出占财政全部支出75%以上，全市社会保险参保总人数达到386万人（次），全民参保目标基本实现。城镇、农村常住居民人均可支配收入分别增长8%、7.8%。4万多群众搬进棚改新房。社会大局保持稳定，连续7年被评为全省综治优胜单位。

一年来，我们主要做了以下五个方面的工作：

（一）调结构。

优化产业结构。推动产业提升价值链、产品迈向中高端，有色金属、黑色金属、建材产业保持两位数增长，电子信息、生物医药等四大产业增长20%以上，其中高端装备制造增长实现翻番。突破性发展现代服务业，海尔日日顺物流园建成运营，新增3A级以上物流企业6家，完成物流业增加值75亿元。新设立光大银行黄石分行、开源证券黄石营业部。引进阿里巴巴、火凤凰等12家知名电商企业，阳新入选中国电商示范百佳县，居然之家等重大项目加快推进。

优化投资结构。聚焦投资质量和效益，工业投资增长19.3%，占固定资产投资比重达43%。实施重点工业技改项目208个、总投资540亿元。开展“双千”活动和民营经济“暖冬行动”，出台支持民营经济发展一揽子政策，优化营商环境，增强投资信心，民间投资增长12%。围绕“五城”建设、推进高质量发展，加强项目谋划，建立了总投资过万亿元的储备库和建设库。

优化城市空间结构。按照东拓南进西融的思路，把大冶湖新区作为未来的城市中心，完善路网，拓展骨架，打通血脉，带动新港物流（工业）园、黄石开发区、大冶湖高新区联动发展。对接顺丰机场，积极谋划临空经济区。推进新港物流（工业）园沿江布局，成立海口湖新区，港口后方陆域发展空间进一步拓展。

（二）换动能。

以创新换动能。出台建设创新活力之城的政策措施，筹备设立10亿元（首期2亿元）高新技术产业发展引导基金。大力实施“四个十”科创工程，新组建板带新材料、化学药物产业研究院，新认定省级企业技术中心4家。完成省级科技成果转化80项、专利授权量2348件。远大生科获批中国驰名商标，新增湖北名牌产品13个。

以改革强动能。在全省率先推行“先建后验”、“三十七证合一”、“证照分离”、招投标“评定分离”等改革，“智慧黄石”政务平台上线运行。积极推进政企、事企分离、“三供一业”分离，20家行政事业单位所属企业重组到市级投融资平台，15家中央、省属企业物业移交地方管理。加强社会领域改革，获批国家第三批社区居家养老服务、首批守信激励创新试点城市。调整设置城区街道办事处。652个村完成农村集体产权制度改革。

以开放增动能。黄石新港一期后方陆域堆场、散货转运设施全面建成投产，近洋直航、江海直达始发航线实现常态运行，全市港口完成吞吐量4200万吨。着力提升通关便利化水平，口岸整体通关时间压缩1/3。引导企业参与“一带一路”建设，新增外贸出口企业48家，华新水泥塔吉克斯坦、柬埔寨项目入选全省“一带一路”重点支持项目库。

（三）转方式。

提高全要素生产率。优化金融、人才、土地等要素配置，推动生产要素向主导产业集中。黄石连续14年被评为全省金融信用市，市级投融资平台新增融资195亿元。坚决落实减税降费政策，为企业减轻负担12亿元以上。实施“新黄石人”计划，发放稳岗补贴3788万元，帮助企业招工3.8万人次。处置闲置土地39宗、1027亩，消化批而未供土地1.02万亩。

提高产业集中度。推进工业企业“退城入园”，启动华新水泥4、5号窑关停和新兴铸管、宝钢薄板搬迁。加快特色园区建设，西塞山智能模具和模具材料、铁山工模具等园区渐成规模。狠抓产业链招商，新签约重点项目178个、总投资1284亿元，成功引进信濠光电、宏和电子等重大项目，全国第三大PCB产业聚集区地位进一步巩固，黄石开发区被授予海峡两岸产业合作示范园。新港物流（工业）园列入国家第三批增量配电改革试点，新港新材料产业园启动建设。狠抓项目调度，新开工、新投产亿元以上项目分别达到140个和162个。

提高城市文化竞争力。以创建国家历史文化名城为抓手，启动申遗价值研究，确定市级第一批工业遗产名录，西塞山区获批中国端午神舟文化之乡，铜绿山古铜矿遗址博物馆新馆基本建成。铁山区创建国家全域旅游示范区稳步推进。大力发展文化旅游产业，新签约帆船小镇、青港湖欢乐世界等项目，开工建设楚天香谷田园综合体，建成南市渔歌乡博园，完成旅游综合收入169亿元、增长20%。

（四）促协调。

促进区域协调。坚持以乡村振兴为抓手，着力推进 “四化”同步、城乡统筹。新组建涉农产业联盟7个，稻虾共作、柑橘种植面积分别超过10万亩、8万亩，大冶市获批全国休闲农业示范县、创建三产融合发展先导区。新改建农村户厕42510户、公厕1346座。灭荒造林7.3万亩。整治高标准农田10.7万亩，实施水利工程338处、投资8.75亿元。棋盘洲、武穴长江大桥及连接线等项目顺利推进，建成一二级公路73公里，新改建农村公路439公里，完成交通投资118.5亿元，大冶市荣获“四好农村路”全国示范县称号。

促进新老城区协调。以承办省运会为契机，加快新区建设，奥体中心“一场两馆”、奥林匹克公园投入使用，城市综合馆、市民之家基本完工，市社会福利中心、园博园二期顺利推进，大冶湖核心区“惊艳亮相”。促进老城提质，完成铜都社区等10个老旧小区改造，上窑轮渡农贸市场关闭、分流；完成湖滨大道、广州路、大泉路、马鞍山路等改造升级，建成黄石新百等5座人行天桥，维护改造城市路面5.6万平方米；建成口袋公园25个，新增和改造城市绿地79公顷。

促进经济社会协调。坚持创业带就业，新增省级科技企业孵化器2家、众创空间1家，扶持成功创业6983人、带动就业2.4万人。加大社会保障力度，市财政安排3.9亿元补充企业职工养老金缺口。优化义务教育资源供给，武黄路小学、钢山学校等项目启动实施。统筹推进“新高考”、职业教育、高等教育改革，教育教学质量不断提高。有序推进医改工作，“三医联动”机制初步形成，城区三级公立医院人均住院费用下降10%。“村村响”和“户户通”工程通过省级验收，新群艺馆、西塞山公共文化服务中心等建成开放，阳新采茶戏《龙港秋夜》作为我省唯一曲目，参加全国基层院团戏曲会演。

（五）固底板。

坚决向污染宣战。实施长江大保护“十大标志性战役”和绿色发展“十大战略性举措”。深入开展“水气山土”治理，全面落实河湖长制，狠抓城市黑臭水体治理，新建和改造市区污水管网78公里，完成7座污水处理厂一级A提标改造，全市乡镇污水处理厂及管网基本建成，餐厨垃圾处理厂投入运行，全市国控、省控跨界考核断面和县级以上饮用水源地实现Ⅲ类水质达标，PM2.5、PM10同比分别下降20.8%和15.9%，空气优良天数超过270天，“三区两线”矿山塘口治理全面启动，东钢地块等8个土壤治理项目加快推进。

全力抓好精准扶贫。聚焦“两不愁三保障”，深入实施“一户一策、一村一法”， 22个村出列、4.67万人脱贫。市级财政安排1.15亿元支持阳新县脱贫摘帽，发放小额扶贫信贷3.78亿元、惠及贫困户8953户，发展“五当家”产业项目1万多个，易地搬迁785户、1019人，危房改造1160户，就业培训4041人，资助贫困学生3.6万人，发放农村低保金1.6亿元、惠及4.7万人。

切实防范化解重大风险。严格政府债务预算管理和投资项目审批，政府债务负债率、市级投融资平台资产负债率均低于控制线。着力防范化解社会风险，深入开展“打非治违”专项行动，加强矿山、冶金、危化品、建筑及道路交通专项治理，全市安全生产形势总体平稳。扎实开展扫黑除恶、扫黄打非等专项斗争，着力化解信访矛盾。

始终把坚持党的全面领导作为最高政治原则，狠抓政府自身建设。切实提高政府工作政治站位，树牢“四个意识”，坚定“四个自信”，坚决做到“两个维护”，认真做好中央及省环保督察、省政府大督查反馈问题整改。加强法治政府建设，有序推进政府立法，扎实做好政府投资项目审计监督，自觉接受市人大及其常委会的法律监督和工作监督，主动接受市政协的民主监督。加强应急处置能力建设，成功处置东方山水库渗漏重大险情。聚焦重点工作、重点项目，坚持正向激励和反向惩戒相结合，为154家单位、503人次记功嘉奖，对16家单位实行挂牌警示通报。

与此同时，统计、外事、侨务、民族、宗教、对台、保密、档案、妇女儿童、老龄、慈善、援藏、援疆、国家安全、国防动员、民兵预备役、广播电视、新闻出版、民防、水文、气象、史志等各项工作均取得新成绩。

各位代表！成如容易却艰辛。过去的一年，外部环境之变、风险挑战之多、转型发展之难，均超过预期，能够取得这样的成绩实属不易。这是省委省政府和市委正确领导的结果，是市人大、市政协监督支持的结果，也是全市人民团结奋斗的结果。在此，我代表市人民政府，向全市人民，向各位人大代表、政协委员，向各民主党派、各人民团体和各界人士，向驻黄解放军、武警官兵，向所有关心支持黄石建设发展的海内外人士，表示衷心的感谢！

各位代表，前进路上无坦途。我们清醒地认识到，黄石发展还面临着不少困难和挑战：受中美贸易摩擦影响，经济运行稳中有变、变中有忧，外部环境复杂严峻，经济发展不确定因素增多；经济下行压力加大，稳的基础还不牢固，实体经济尤其是部分民营企业、中小微企业运行困难；新旧动能转换较慢，经济结构还不优，经济发展韧性还不强；民生工作不平衡、不协调的矛盾比较突出；政府效能有待进一步提高，少数干部怕问责、不担责现象依然存在，等等。对这些发展中的困难、前进中的问题、成长中的烦恼，我们将采取有力措施，切实加以解决。

二、2019年工作的总体要求和主要目标

各位代表，凡是过去，皆为序章。今年是建国70周年，是改革开放再出发之年，是决战决胜全面建成小康社会的关键之年，做好今年的政府工作，意义重大，任务艰巨。

今年政府工作的总体要求：以习近平新时代中国特色社会主义思想为指导，全面贯彻党的十九大和中央经济工作会议、省委十一届四次全会暨经济工作会议精神，坚持稳中求进工作总基调，坚持新发展理念，深化供给侧结构性改革，落实省委省政府“一芯两带三区”区域和产业发展战略，按照市委十三届七次全会的部署，以打好三大攻坚战、推动高质量发展为主题主线，围绕“五大目标”，推进“五大转型”，持续保持当前我市经济社会发展良好态势，为全面建成小康社会打下决定性基础，在鄂东转型发展示范区中发挥引领带动作用，以优异成绩向新中国成立70周年献礼。

经济社会发展的主要预期目标：地区生产总值增长7.5%-8%；规模以上工业增加值增长8%以上；固定资产投资增长11%左右；社会消费品零售总额增长11%左右；外贸出口增长6%左右；地方公共预算收入增长7%左右；居民消费价格指数控制在3.5%左右；城镇登记失业率控制在4.5%以内；城乡居民收入与经济增长基本同步；单位生产总值能耗下降2.5%；完成省定主要污染物减排任务。

全面建成小康社会综合指数达到92%以上，其中服务业增加值占GDP比重提高1个百分点，研究与试验发展经费投入强度达到2%，高新技术产业增加值占GDP比重达到18%，文化产业增加值占GDP比重达到2%。

围绕以上目标要求，我们将着重把握以下六点：

一是坚持稳中求进的工作总基调。着重把握好稳和进的关系，既要聚焦“六稳”夯实底盘，也要以进促稳乘势而上。坚定信心，坚定不移办好自己的事，坚决保持住、维护好当前我市发展逆势而上的良好势头,牢牢掌握发展主动权。

二是深入推进“一芯两带三区”区域和产业发展战略实施。围绕“一芯驱动”，强产业之“芯”，建区域之心，育动能之“新”，在武黄融合、多极支撑上下功夫；围绕“两带支撑”，在绿色环保、创新驱动上做文章；围绕“三区协同”，在转型升级、示范引领上有新作为。

三是突出打好三大攻坚战、推进高质量发展的主题主线。坚持应急与谋远相结合、质量与速度相统一，既抓重点、又谋全局，既补短板、又固底板，既讲拼劲、又有韧劲，始终抓住主题主线，针对突出问题，打好重点战役，为全面建成小康社会打下决定性基础。

四是紧紧扭住“五城”建设的战略目标。坚持“项目为王”，把着力点放在动能、产业、功能、生态、空间“五个转型”上，把支撑点落实为一个个优质产业、具体项目，加快建设创新活力之城、先进制造之城、现代港口城市、山水宜居之城、历史文化名城。

五是坚持把长江大保护作为压倒性任务。系统谋划生态保护、绿色发展和乡村振兴，突出“山水林田湖草”综合治理，深入开展“碧水、绿岸、洁产、畅流”四大行动，奋力推动黄石生态环境根本好转，实现老工业城市的完美转身。

六是牢固树立以人民为中心的根本立场。按照幼有所育、学有所教、劳有所得、病有所医、老有所养、住有所居、弱有所扶的要求，从群众最急最忧最盼的事情办起，从老百姓反映最强烈的就学看病出行等难题抓起，一件事情接着一件事情干，一锤接着一锤敲，让我们的城市更有温度，让人民群众生活得更体面、更幸福,更有安全感、归属感和自豪感。

三、2019年工作重点任务

（一）突出实体经济，着力推进经济稳健增长。围绕“六稳”要求，坚持问题导向，突出实体经济、有效投资、营商环境,综合施策、精准发力，推动经济行稳致远。

着力服务实体经济。聚焦民营企业和中小微企业融资难、用工难、转型难，深入开展“双千”活动，努力帮助企业纾困解难。进一步提高市担保集团担保能力，加快建立“4321”风险分担模式，大力推行无还本续贷、纳税信用贷、应收账款融资等业务。大力弘扬工匠精神，深入实施“新黄石人”计划，探索专家返聘、项目引才、人才租赁、候鸟专家、科技特派员等柔性引才方式，力争引进培育高端人才、技能人才、产业工人2万人。用好用活转型帮扶资金、产业基金，支持民营企业开展技改、研发、兼并重组，实现转型升级、做优做强。深入实施中小企业成长工程，力争新增规模以上工业企业60家。

千方百计扩大有效投资。坚持以项目稳投资，力争全年新开工亿元以上项目150个，建成投产100个。健全项目调度拉练机制，重点抓好十大技改、十大工业招商、十大服务业、十大交通基础设施、十大公共服务设施、十大文旅项目“六个十”工程。坚持以招商扩投资，深入推进专业招商、精准招商、产业链招商和以商招商，力争新注册重点项目170个以上，实际到位内资1260亿元，实际利用外资增长7%。抢抓国家重大政策机遇期和窗口期，精心策划、做实落地一批重大项目。

优化营商环境。强化政策预期，聚焦金融、用工、能源、环保、审批等问题，坚持小切口、能感知、有温度，积极落实支持中小微企业、民营企业发展的各项政策措施，形成强有力的政策组合拳。帮助企业有效应对中美贸易摩擦影响，积极开拓新兴市场。落实优化营商环境“60条”，坚持“无事不扰，有求必应”，严禁监管执法“一刀切”，鼓励引导企业规范经营，增强市场主体投资信心。大力弘扬企业家精神，切实营造尊商、重商、爱商、护商的浓厚氛围。

（二）突出先进制造，着力推进产业转型升级。以国家产业转型升级示范区建设为抓手，推进制造业高端化、集群化、智能化、绿色化、融合化发展。

大力发展先进制造业。深化供给侧结构性改革，通过技术改造、招商引资、兼并重组，重点发展新材料、新能源、电子信息、大健康等九大主导产业，重塑我市产业竞争新优势。加快实施新港重科、新冶钢产能置换、华中铜板带箔、铜铝加工产业园、华新百年复兴基地等重点项目，推动我市由原材料基地向新材料基地转型。加快推进信濠光电、汉龙汽车零部件产业园、铁流汽车零部件等重点项目，努力打造全国电子信息基础产业基地、智能装备产业基地。加快实施劲牌健康基地、世星药业二期、大冶有色多基固废清洁生产、博天中环膜等重点项目，努力打造全国大健康产业基地、节能环保产业基地。着力培育一批隐形冠军企业，新增高新技术企业50家，高新技术产业增加值增长10%。

推动制造业集群发展。以黄石开发区、大冶湖高新区、新港物流（工业）园为主战场，创新投入机制，完善水电气路管网等配套设施，优化产业布局，促进集群发展。深入推进海峡两岸产业合作示范园建设。加快模具钢、智能传输等七大特色产业园建设，实现集聚、错位、协同发展。支持企业以“退城入园”为契机，推进产能、装备、技术全方位升级。

推进先进制造业与现代服务业融合发展。按照“先进制造+现代服务”的思路，大力发展现代物流、现代金融、研发设计、检验检测、信息服务、技术咨询等生产性服务业，推动新技术、新产品、新业态、新模式蓬勃发展。积极开展“两化”融合贯标和智能制造试点，推动实体经济与互联网、人工智能深度融合。鼓励纺织服装等消费品工业利用互联网平台深挖市场需求，开展个性化产品定制。放大中国工业互联网创新大会永久会址的品牌效应，着力引进一批互联网、智能制造、电子信息等关联企业，努力打造全国工业互联网产业创新发展先行区。

（三）突出改革创新，着力增强发展活力。以创建国家创新型城市为抓手，以科技创新为核心，优化要素资源配置，打造创新活力之城。

强化科技创新核心作用。坚持将创新链植入产业链，提升价值链，推动“三链”融合，深入推进“四个十”科创工程，开工建设黄石科技城和科创中心，积极推动产学研合作，新增市级以上研发机构65家，加快实现主导产业技术研究院、重点企业技术研发中心全覆盖。加强众创空间、孵化器等“双创”平台建设。组建市高新投公司。强化知识产权保护与运用，新增发明专利授权量150件，进一步提高科技成果转化水平。大力实施增品种、提品质、创品牌工程，新增地理标志2件、湖北名牌产品10个，力争成功创建全国质量强市示范市。

推进要素高效配置。围绕主导产业、重点项目配置资源。大力发展科技金融、绿色金融、普惠金融，扩大直接融资，启动东贝B股转A股，支持航天电缆等企业科创板上市。加快市级投融资平台市场化改革，力争实现融资200亿元。坚持以亩产论英雄，探索推进工业项目“标准地”改革，推行工业用地租赁和弹性出让；实施城镇低效用地再开发，深化增减挂钩试点，着力盘活闲置土地。

深化重点领域改革。加快推进“一网一门一次”改革，实现政务服务一张网“五级联通”。将“先建后验”试点拓展到省级园区工业投资项目，持续促进审批提速。深化商事制度改革，激发微观主体活力，力争市场主体总量突破22万户、增长7.5%。深化财政改革，全面实施预算绩效管理制度，加强和规范行政事业单位经营性资产管理。深化国资国企改革，大力引进央企、有实力的民企、战略投资者参与重组，推进混合所有制改革，落实国有资产报告制度，促进从管企业向管资本转变。深化公立医院、“三医联动”改革，加快推进医共体建设，推动医疗资源向基层下沉。

（四）突出以港兴城，着力提升开放水平。以黄石新港为龙头，以基础设施为重点，加快推进水港、空港、铁路港、公路港“四港”联动、协同发展，提升城市枢纽地位。

建设大港口。深入推进新港“亿吨大港”建设，建成新港码头二期，开工建设新港码头三期、战备滚装码头、黄颡口砂石集并中心；加快推进沿江大道、河金省道改造，建成新港大道北延、海州大道北延、棋盘洲长江大桥连接线，完善新港集疏运体系。坚持全域对接、交通先行，扎实做好对接顺丰机场“1+6”快速通道、黄石通用机场前期等工作，积极融入武鄂黄黄协同发展战略，共同打造国家级空港经济综合试验区。加快推进山南铁路适应性改造，建成新冶钢铁路支线，实施黄石北站改扩建工程。基本建成传化公路港。

构建大路网。完成交通投资120亿元，加快构建外联内畅的综合交通体系。加快推进武阳高速黄石段、鄂咸高速黄石段、棋盘洲和武穴两座长江大桥建设，蕲嘉高速大王至金湖段建成通车，着力完善高速公路网。加快实施铁山至下陆一级公路项目。推进港湖至茅圻、大田至三溪、木港至龙港、刘仁八至金牛、保安至毛铺等国省干线改造。

发展大物流。科学谋划物流链、供应链、产业链布局，加快推进棋盘洲综合物流园、捷利物流园、粮食物流园建设，建成运营润信农批物流园、超捷物流园，支持有色物流公司创建国家5A级物流企业。延伸物流、金融、仓储、展示、交易等电商产业链，支持大道众采等企业做大做强，筹建跨境电商信用保障资金池，打造全省跨境电商先行示范区。

推进大通关。建立口岸服务联动机制，将口岸监管区拓展覆盖到铁路港，实现铁水通关一体化。加快申报综合保税区、进境粮食指定口岸、启运港退税试点口岸。充分发挥新港多式联运示范线路作用，优化江海直达和黄石至成都货运班列，再压缩进出口通关时间10%以上，进一步降低货物通关运输综合成本，全面提升开放水平。

（五）突出新区引领，着力推进全域一体发展。以大冶湖新区为龙头，坚持新区老城、城市乡镇两手抓，促进城乡统筹、全域融合。

新区建设全面提速。实施沿湖公路及生态景观工程，延伸园博大道、奥体大道连接大冶城区，推进有轨电车连通新老城区。坚持“新业态、新生活、新居住、新社区、新服务”理念，开工建设绿地中心城、月亮湾新城、黄石教育城、邻里中心等项目，建成黄石市中心医院黄金山院区，狠抓水电气、热能、管廊、公共交通、防洪设施等配套完善，着力引进大型商业、游乐项目，加快人口向新区转移，努力把新区建成黄石的科创中心、会展中心、文旅体育中心、行政副中心以及城市会客厅，让新区更有吸引力。

老城改造全面提升。按照统一规划、试点先行的思路，着力推进102个社区环境综合整治。深入推进棚改新三年计划，开工棚改项目17个、10572户，基本建成17350户。坚持补短板、强功能，加快磁湖南岸路网建设，推进26条道路微循环改造，实施智慧停车项目，增加人行天桥，着力解决出行难、行车难、停车难问题。开工建设环磁湖绿道、黄荆山城市公园，建成磁湖湿地公园二期。坚持标本兼治，新建城区二三级污水管网60公里，基本实现雨污分流。

城乡融合全面加快。坚持全域统筹发展，推动中心城区与县城、重点镇对接融合，完善新型城镇化体系。按照聚人口、能就业的思路，突出产业支撑，加快城镇路网管网建设，完善公共服务设施，吸引更多农民就地就近城镇化。

（六）突出城市创建，着力提升城市魅力。坚持城市创建与功能转型相结合，城市文化与产业发展相融合，增强城市软实力。

彰显城市文化内涵。深入实施创建国家历史文化名城三年行动方案，大力推进历史建筑保护修缮、特色场馆建设等八大工程，突出抓好华新水泥旧址、东钢旧址、大冶钢厂苏式建筑群、铁山光明里、黄石港地质里等历史文化街区建设，加快实施卸矿机展示利用等文保项目，积极推进矿冶工业遗产申遗。传承和保护大冶刺绣、阳新采茶戏、阳新布贴、西塞神舟会等非物质文化遗产，培育2个省级特色文化小镇，创建4个省级以上传统村落。

加快商旅文融合发展。坚持发展全域旅游，以工业旅游为重点,支持基础较好的厂矿、园区实施景点改造，建设标准化观光工厂，引进知名文创企业进驻华新、东钢文创园，让生产园区、工业遗址可进入、可体验、可互动。大力发展地矿科普、教育研学等产业，推进矿博园特色国际馆建设，推动工业旅游专线火车市场化运营。积极培育新零售、体验经济等消费新业态新模式，加快推进大型商业综合体建设。

加强城市精细化管理。以创建全国文明城市为抓手，健全路长制、社区包保等长效机制，提升市民文明素质和城市建设管理水平。加强城管联合执法，开展交通秩序、市容市貌、城市养犬、“门前三包”等专项整治。加快建筑垃圾资源化利用，推行生活垃圾强制分类处置。科学布局一批新能源汽车充电桩，规范发展网约车，实行共享单车“电子围栏”管理。强化城市天际线、建筑立面、色彩设计管控和植被整理，提升城市品位。

（七）突出乡村振兴，着力加强“三农”工作。围绕产业、人才、文化、生态、组织 “五个振兴”，加快推进农业农村现代化。

大力发展现代农业。深化农业供给侧结构性改革，推动农业规模化、链条化、特色化发展。加快实施“双十双百”工程，着力打造楚天香谷、稻梦空间等10个都市田园综合体，培育金牛、富池等10个三产融合发展示范镇，建设“双水双绿”2个百万亩特色农业基地。加快黄石国家农业科技园建设，设立农业产业发展基金，支持专业合作社、家庭农场、农产品加工龙头企业发展。扎实开展农产品质量安全追溯及“三品一标”建设。鼓励发展互联网+现代农业+旅游，着力打造赏花、采摘等农旅节庆品牌。

持续开展乡村环境整治。深入实施“四个三重大生态工程”，加强农业面源污染综合治理，大力开展“三清一改”村庄清洁行动，推广运用人工湿地、微动力生化等技术收集处理农村生活污水，推进农村生活垃圾无害化处理。加快实施“四大补短板工程”，完成“四好农村路”200公里，建设高标准农田5万亩，整治病险水库30座，解决农村2.6万人安全饮水的提档升级问题。大力开展农村宗族祠堂、教堂及“大棚房”专项整治，有效发挥无职党员、返乡创业能人和农村乡贤作用，推动移风易俗，树立文明新风。

全面深化农村改革。深入实施“三乡工程”，推动城市资源要素向农村流动。积极稳妥推进农村承包地“三权分置”改革，规范发展土地托管合作社，有效盘活闲置农房和宅基地，促进土地经营权可流转、受保护、能抵押。深入推进农村集体产权制度改革，抓好供销社综合改革和农场企业化改革。

（八）突出重点战役，着力推进三大攻坚战。紧盯问题，持续用力，为高质量发展“拔钉清障”。

推进污染防治攻坚战。深入实施“碧水、绿岸、洁产、畅流”行动，扎实推进长江大保护“十大标志性战役”和绿色发展“十大战略性举措”，以沈家营码头、上窑码头、阳新黄颡口至富池段综合治理为重点，坚持江堤路景、岸线修复、城市整理、塘口治理、村庄整治相结合，努力再现水清、岸绿、村庄美的江南风貌。加快16家沿江化工企业关改搬，加快磁湖、大冶湖、富河等重点水域污染治理修复，狠抓城区污水处理厂扩能提标和黑臭水体整治。完成铁山—还地桥矿山地质环境恢复治理等示范工程，在产绿色矿山创建率达到100%。持续推进土壤污染防治先行区建设。坚决打好蓝天保卫战。支持开展环保公益诉讼。完成灭荒造林4.5万亩，巩固提升国家森林城市创建成果。

推进精准脱贫攻坚战。积极做好中央扶贫专项巡视整改，确保通过国家、省扶贫开发成效考核及贫困县退出专项评估。下足“绣花”功夫，重点解决影响“两不愁三保障”的突出问题，确保在脱贫攻坚期内，摘帽不摘责任、摘帽不摘政策、摘帽不摘帮扶、摘帽不摘监管，巩固提升全域脱贫成果。大力发展桑蚕、苎麻、油茶、腊米、白茶等富民产业，切实把贫困户嵌入产业链，完善贫困户增收机制，统筹推进行业扶贫，确保实现长效稳定脱贫。

推进风险防控攻坚战。按照坚定、可控、有序、适度的原则，规范举债行为，加强政府债务预算管理。引导企业适度负债、积极化债、诚信经营，守住信用底线。严厉打击各类非法金融活动，妥善处理涉众型金融案件，切实防范化解金融风险。加强市场调控和稳定预期，促进房地产市场持续健康有序发展。坚决遏制重大交通事故、突发环境事件、群体性事件发生，积极防范化解公共安全、网络安全、社会稳定等领域风险。

（九）突出以人为本，着力保障和改善民生。坚持尽力而为，量力而行，坚持问题导向，实实在在为群众解难题、增福祉。

提升就业和社会保障水平。全面落实稳就业的各项政策，支持高校毕业生、务工返乡人员等重点群体创业就业，探索重点产业用工联盟机制。实施全民参保及社保共享计划，推进社保扩面征缴，完成社会保险费征管职责划转。保护妇女儿童、老年人、残疾人合法权益。建成市社会福利中心。

推进基本公共服务提质。坚持旧城改造、新区开发与学校建设统筹考虑，大力实施基础教育布局专项规划，加强普惠性幼儿园建设，加快实施白马山小学、黄石三中等“改扩搬”项目，落实普及高中阶段教育计划，支持在黄高校“双一流”和应用型本科建设，深化职业教育产教融合，支持和规范民办教育发展。加强“全面二孩”配套服务，促进优生优育。加快互联网+医疗健康服务体系建设，扎实开展国家社区居家养老服务改革试点，推动医疗卫生与养老服务深度融合。实施文化惠民工程，加强基层综合文化服务中心建设，举办第十五届全国地方版图书博览会。加强“后省运”体育事业和产业发展，鼓励市场主体举办各类体育赛事，加快足球青训基地、水上运动基地等项目建设，支持“足球进校园”，鼓励乒乓球职业化发展，广泛开展群众体育活动。

创新和加强社会治理。深入开展扫黑除恶专项斗争，大力实施“雪亮工程”，深化拓展治安防控体系“九张网”，加快智慧警务及综治中心建设。完善自然灾害、公共安全和生产安全事故应急处置预案，进一步增强应急处置能力。深入推进社会信用体系建设。加快建设公共法律服务平台及行业调解委员会，依法化解信访矛盾纠纷。加强市场秩序监管。加强国防动员建设，积极做好退役军人事务和“双拥”共建工作，积极创建全国“双拥”模范城。开展第四次全国经济普查。启动“十四五”规划研究。

各位代表，打铁必须自身硬。我们将按照“五个过硬”要求，努力建设人民满意的服务型政府。

以学习增本领提能力。坚持用习近平新时代中国特色社会主义思想武装头脑、指导实践、推动工作，坚持和加强党的全面领导，树牢“四个意识”，坚定“四个自信”，做到“两个维护”，增强“八种本领”。深入推进学习型政府建设，不断提升应对复杂局面和科学施政、为民理政的能力水平。

以改革转职能提效能。按照中央和省市委统一部署，全面推进政府机构改革，努力建设权责明晰、运转高效、优化协同的服务型政府。建成市民之家，提高“12345”公共热线平台服务能力，优化政务服务流程，努力做到程序简约、管理精细、时限明确，最大限度便民利企。

以担当转作风抓落实。全面落实中央八项规定及实施细则精神，坚持项目为王、干事为先，坚决执行市委进一步改进工作作风、激励担当作为的规定，着力解决“六多”问题，充分调动干部干事创业的积极性、主动性和创造性。认真开展调查研究，坚持务实重行、高效执行，坚决反对表态多、调门高、行动少、落实差，推动中央和省市委决策部署落地生根。

以清廉扬正气树形象。认真履行全面从严治党主体责任，进一步加强党风廉政建设。坚持依法行政，完善政府立法和法律顾问制度，自觉运用法治思维和法治方式推动工作。主动接受市人大及其常委会的法律监督、工作监督，自觉接受市政协的民主监督，认真办理人大代表建议、政协委员提案，认真听取民主党派、工商联、无党派人士和各人民团体意见。全面加强审计监督。认真做好政务公开、新闻发布工作，主动接受社会和舆论监督。大力弘扬契约精神，承诺的事坚决兑现，切实增强政府公信力。

各位代表，时代出卷，我们答卷，人民阅卷。让我们更加紧密地团结在以习近平同志为核心的党中央周围，高举习近平新时代中国特色社会主义思想伟大旗帜，在市委的坚强领导下，融旧铸新，砥砺奋进，努力交出让人民满意的答卷，以优异成绩迎接新中国成立70周年！
